# Diagnostic significance of rib series in minor thorax trauma compared to plain chest film and computed tomography

**DOI:** 10.1186/1752-2897-8-10

**Published:** 2014-08-05

**Authors:** Patrick Hoffstetter, Christian Dornia, Stephan Schäfer, Merle Wagner, Lena M Dendl, Christian Stroszczynski, Andreas G Schreyer

**Affiliations:** 1 Radiology, Asklepios Medical Center, Bad Abbach, Germany; 2 Radiology, University Medical Center, Regensburg, Germany

**Keywords:** Minor thorax trauma, Conventional radiography, Rib series (RS), Computed tomography (CT)

## Abstract

**Background:**

Rib series (RS) are a special radiological technique to improve the visualization of the bony parts of the chest.

**Objectives:**

The aim of this study was to evaluate the diagnostic accuracy of rib series in minor thorax trauma.

**Methods:**

Retrospective study of 56 patients who received RS, 39 patients where additionally evaluated by plain chest film (PCF). All patients underwent a computed tomography (CT) of the chest. RS and PCF were re-read independently by three radiologists, the results were compared with the CT as goldstandard. Sensitivity, specificity, negative and positive predictive value were calculated. Significance in the differences of findings was determined by McNemar test, interobserver variability by Cohens kappa test.

**Results:**

56 patients were evaluated (34 men, 22 women, mean age =61 y.). In 22 patients one or more rib fracture could be identified by CT. In 18 of these cases (82%) the correct diagnosis was made by RS, in 16 cases (73%) the correct number of involved ribs was detected. These differences were significant (p = 0.03). Specificity was 100%, negative and positive predictive value were 85% and 100%. Kappa values for the interobserver agreement was 0.92-0.96. Sensitivity of PCF was 46% and was significantly lower (p = 0.008) compared to CT.

**Conclusions:**

Rib series does not seem to be an useful examination in evaluating minor thorax trauma. CT seems to be the method of choice to detect rib fractures, but the clinical value of the radiological proof has to be discussed and investigated in larger follow up studies.

## Introduction

Conventional rib series (RS), also called oblique views, are a very common routinely performed radiological examination in any radiological department. It represents a special radiographic technique to visualize the bony parts of the chest wall by one or more oblique views, with or without marking the points of maximum tenderness. Comparing with plain chest films the examinations are acquired with lower x-ray energy and a smaller field of view to optimize the bony contrast of the ribs. Especially for trauma patient this technique is requested routinely in the emergency room (ER) to visualize rib fractures. Severe thorax trauma is regularly evaluated by multidetector computed tomography (MDCT), though typically minor blunt traumas are examined by RS [1-4]. Diagnostic accuracy of radiography in evaluating thorax injury is considered to be low compared with computed tomography (CT), but detailed literature data exists only for severe trauma without special focus on rib fractures or dedicated rib series [5-7]. Minor thorax trauma often leads to nothing more than isolated rib fractures without any dislocation. Especially for these cases RS might provide insufficient diagnostic accuracy, as non-dislocated rib fractures cannot always be detected on RS. On the other side these injuries mostly need no further therapy. Therefore, it can be considered irrelevant if a non-dislocated rib fracture escapes detection.

RS are very often combined with plain chest films (PCF), to rule out pneumo- or hematothorax. Both diagnostic strategies are being used, the initial combination of RS with PCF or the consecutive combination. A suspicious bony lesion on PCF could lead to further investigation via RS. On the other hand, in case of a fracture detected on an initial RS, a PCF might be added to rule out possible complications.

The purpose of this study was to reveal the diagnostic significance of RS in patients with minor thorax trauma compared with CT performed in a tertiary care university medical center.

## Material and methods

Based on a retrospective chart review we analyzed all patients who underwent a conventional radiographic examination by RS in our tertiary care university medical center in a two years period between 2008 and 2010. Out of a total number of 767 examinations we identified 56 patients fulfilling the following criteria: [1] indication of examination must be minor blunt thorax trauma (No respiratory distress, conscious patient, no critical condition or vital danger), [2] patients received an additional CT of the thorax, [3] CT must have been performed within two weeks after radiography, and there may be no new trauma. 39 of the 56 included Patients received additionally plain chest film (PCF).

All patients were examined at a digital radiological workplace (Axiom Aristos Multix FDX-Flatpaneldetector, Software VB21B, Siemens Healthcare Erlangen) with 70 kV x-ray energy. Two oblique rib series were performed as grid radiography (Pb15/80) for all patients with a focus distance of 150 cm. Patients were examined in standing position.

All CT scans were acquired with a multidetector spiral CT using a 16-slice CT Scanner (Sensation 16, Siemens Healthcare, Erlangen). Images were acquired using standard settings of 120 kV, 87 mAs, rotation time 0.75 s and a pitch of 1.6. Based on the raw data with a collimation of 16 x 0.75 mm axial and coronal reconstructions were created with a slice thickness of 5 mm and an increment of 4 mm. Image interpretation was performed as soft reading on two high-performance CRT Monitors (Syngo Imaging Advanced, Software Version VB36A, Siemens Healthcare, Erlangen).

In accordance with the local ethics commission and due to the retrospective nature of this study an institutional review board approval was not necessary.

Both, the RS and the CT examinations were read again independently by three experienced board certified radiologists. Firstly, the RS were individually analyzed in a blinded fashion regarding potential rib fractures and associated complications such as pneumothorax. 39 patients received an additional PCF. These examinations were also performed as grid radiography (Pb15/80) in standing position at the same digital working place using 120 KV x-ray energy. These films were evaluated in the same way as the RS. The CT scans were analyzed together by the three radiologists in consensus reading to define the gold standard. The consensus reading of the CT was blinded to the radiographic examinations. The time period between individual and consensus reading was at least two months. In the case of deviations of the original findings and the readers results of the RS and PCF the finding of the majority of the readers was considered as the correct result. The original reports and the results of the readers of the radiographic examinations were compared with the gold standard to define sensitivity, specificity, negative and positive predictive value. The observer variability was calculated using Cohens kappa, the significance between the results of CT, rib series and plain chest film was determined by using the McNemar test. P-values of < 0.05 were considered to be statistically significant.

## Results

56 Patients were included in this study (34 men, 22 women). The mean age was 61 years (range 24-87 years). Considering the initial examination reports of 56 rib series, performed by residents under supervision of a consultant, 15 patients were diagnosed with a rib fracture, while no rib fracture was found in 41 patients. Seven patients had a single rib fracture, three had two broken ribs and 5 had more than two broken ribs. The initial diagnoses were in 98% of the cases confirmed by the second reading performed by the three blinded and independent observers. One patient was diagnosed with a single rib fracture by two of the observers, whereas in the initial report no rib fracture was diagnosed (Table 1). Two observers missed one fracture each so there were at all only 4 deviations from the initial diagnosis by the second reading with a total of 224 diagnoses (4×56). Therefore, we found an almost perfect agreement between the readers and the original report for all readings (Fracture and number of fracture) with high kappa values between 0.89 and 0.96 (Table 2). Two patients with rib fractures had a pneumothorax.

**Table 1:** Findings of rib series and CT of the thorax for n = 56 patients

**No. of fractured ribs**	**No. of patients diagnosed**			
	**Rib series**		**CT**	
0	40		34	
1	7	Total 16	11	Total 22
2	3		4	
> = 3	6		7	

CT = computed tomography; No = number.

**Table 2:** Inter-observer agreement of three readers of rib series for n = 56 patients

**Observer**	**1**	**2**	**3**
Deviations from the initial diagnosis	1	2	1
Kappa value	0,96	0,92	0,96

**Figure 1:**
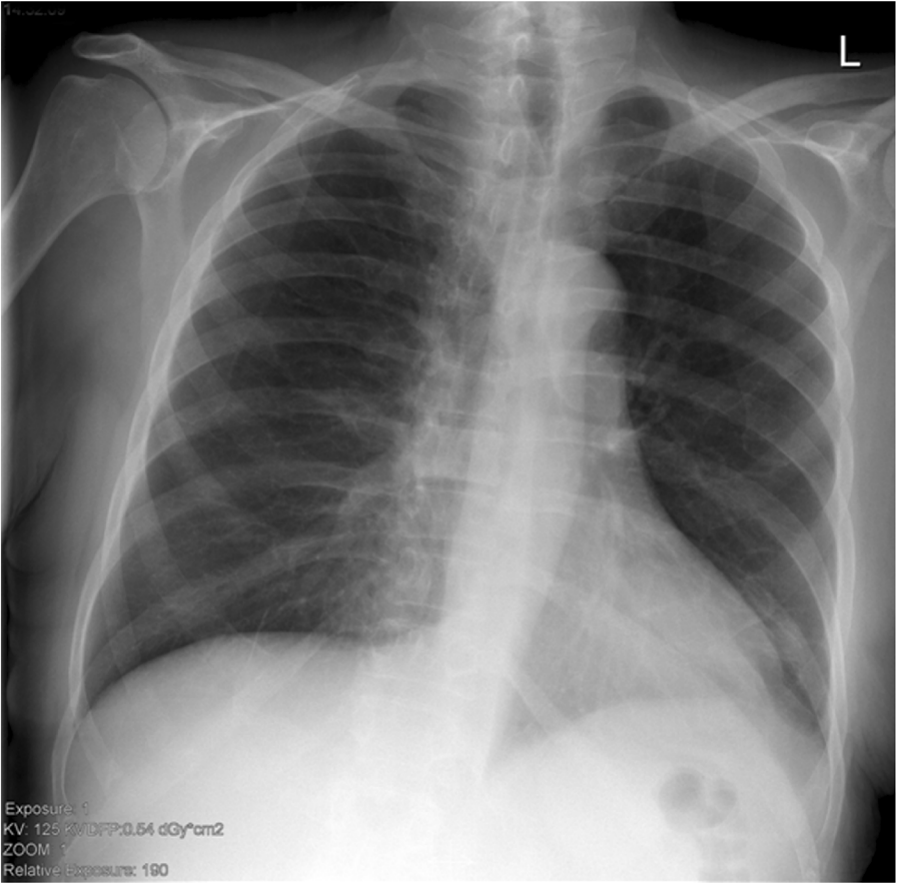
**46-year-old male patient with minor blunt thorax trauma.** No thorax injury is detectable on the chest film.

**Figure 2:**
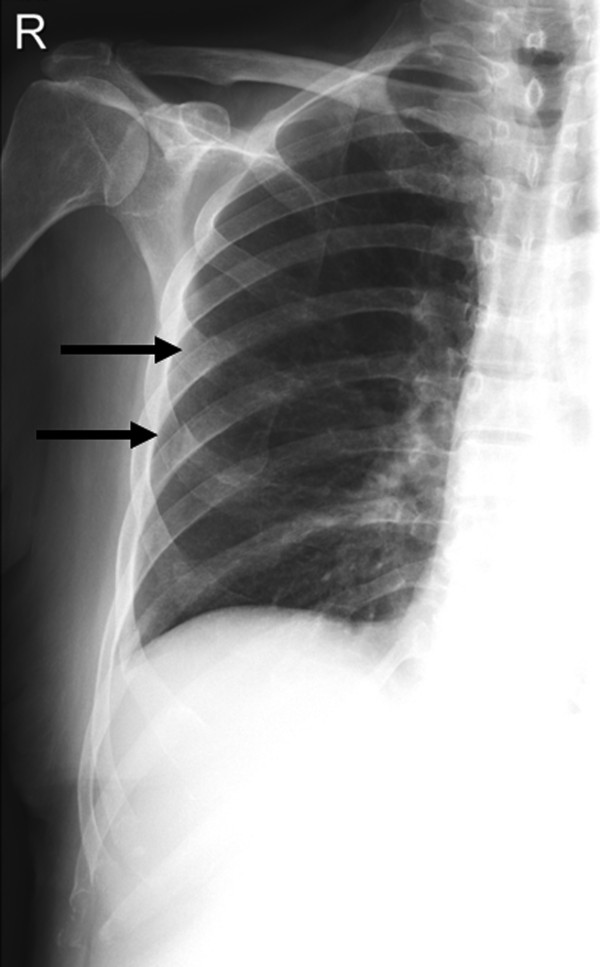
**Same patient as in Figure**1**.** Rib series is showing fracures of rib IV and V on the right side.

**Figure 3:**
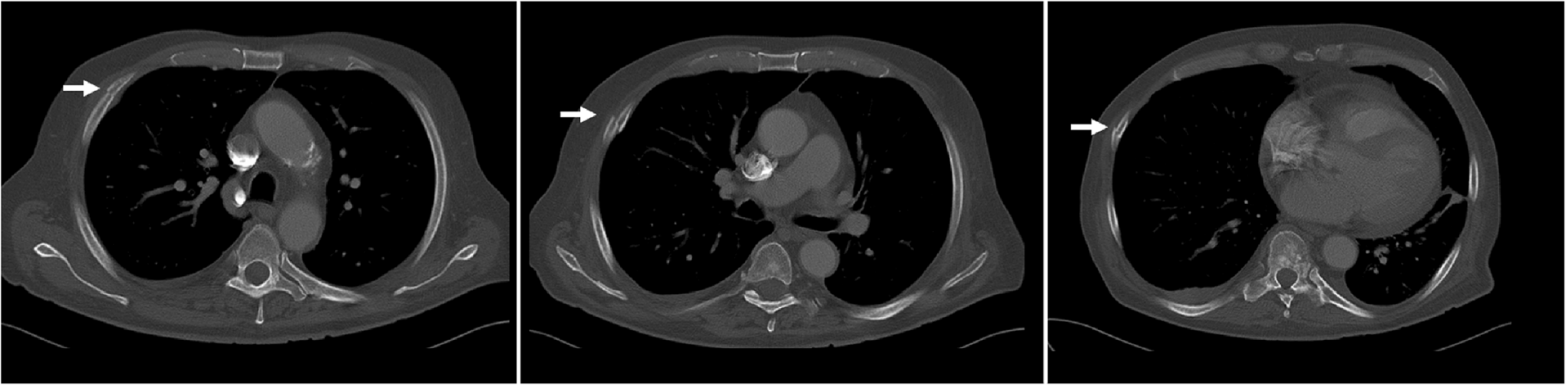
**Same patient as in Figures**1**and**2**.** CT of the chest confirms the fractures of rib IV and V and reveals an additional fracture of rib VII.

The mean delay of the CT scans to the RS was 6 days (Range 0–14) and there was no evidence of an additional trauma during this period. In 22 Patients one or more rib fractures were assessed by CT. 16 of the 22 rib fractures were correctly identified in the rib series including the two patients with pneumothorax. Six additional fractures were missed in conventional radiography but detected in CT. These included 4 cases with a single rib fracture in the CT and a negative result in the rib series, and two patients with three respectively two broken ribs which one broken rib was missed in radiography in each case, which was then detected in the CT scan (Table 1, Figures 1, 2 and 3).

Based on these results a sensitivety of 82% for the presence of rib fractures was calculated comparing the rib series with the goldstandard CT (Table 3). The difference was not statistically significant (p = 0,125, 95% confidence interval: 0.66; 0.98).

**Table 3:** Diagnostic accuracy of rib series comparing with Thorax CT for n = 56 patients

**Rib series**	**CT**	**No**	
			
Fracture	Fracture	18	Sensitivity 82% **p = 0.125 (McNemar)** 95% CI: 0.66; 0.98
No fracture	Fracture	4	
No fracture	No fracture	34	Specificity 100%
Fracture	No fracture	0	

CT = computed tomography; No = number.

Regarding the correct number of fractures the sensitivity of the conventional rib series was 72% (Table 4), which is significant lower compared to CT (p = 0.031, 95% confidence interval: 0.54; 0.91). There were no false positive findings in the rib series either in the original report or in the second reading thus yielding a specificity of 100%. The overall positive predictive value was 100% with a negative predictive value of 85%. Comparing with the 39 patients who received an additional plain chest film, there were 8 false negative findings. In five of these patients the fractures could be diagnosed in the rib series, these differences were not significant (p = 0.063). Comparing with CT the sensitivity of chest film in detecting rib fractures was 46% (Table 5), which was significant lower (p = 0.008, 95% confidence interval: 0.21; 0.72). The specificity of the chest film was also 100%, with no false positive finding. The positive predictive value of chest film was 100% the negative predictive value was 75%.

**Table 4:** Diagnostic accuracy of rib series for detection of correct number of fractures compared with Thorax CT for n = 56 patients

**Rib series**	**CT**	**No**	
Fracture/cor. number	Fracture/cor. number	16	Sensitivity 73% **p = 0.031 (McNemar)** 95% CI: 0.54; 0.91
No fracture/wrong number	Fracture/cor. number	6	
No fracture	No fracture	34	Specificity 100%
Fracture	No fracture	0	

CT = computed tomography; No = number.

**Table 5:** Diagnostic accuracy of chest film comparing with thorax CT for n = 39 patients

**Chest film**	**CT**	**No**	
Fracture	Fracture	7	Sensitivity 46% **P = 0.008 (McNemar)** 95% CI: 0.21; 0.72
No fracture	Fracture	8	
No fracture	No fracture	24	Specificity 100%
Fracture	No fracture	0	

CT = computed tomography; No = number.

## Discussion

Thoracic injury is the third most common result of trauma, following injuries of the head and the extremities [6]. Contrast enhanced CT-Thorax is considered to be the diagnostic approach of choice for severe thoracic trauma. All relevant traumatic diagnoses can be made in short time with high accuracy with CT providing high sensitivity and specificity in detection of pulmonary lacerations, tracheobronchial injuries, pneumothoraces and aortic lesions [8].

The most common thoracic injury are rib fractures which can be found up to 67% of cases with blunt thorax trauma [9]. But rib fractures may also occur without an adequate thorax trauma. Typical risk groups are elder patients suffering from osteoporosis or patients with extreme sports activity such as rowing [10,11].

Fractures are the most common lesion of the ribs. CT offers a high diagnostic accuracy in evaluating the bony parts of the thorax [12]. Although the lower diagnostic sensitivity of radiography compared to CT is a known fact, radiography is established as the method of choice in initial evaluating minor blunt thorax trauma [1]. Plain chest film can depict bony injuries as well as typical complications of rib fractures like pneumothorax and hematothorax. Former studies revealed a sensitivity in detection of rib fractures by chest films of only 15-50% [1,13,14]. CT seems to be superior even in evaluating chest wall injuries.

The low sensitivity of chest radiography may be improved by using rib series with optimized bony contrast. De Lucca examined 100 Patient after thorax trauma with both technics. Chest film revealed 13 patients with rib fracture but rib series detected 28 [15]. As far as we know our study is the first evaluating the diagnostic accuracy of dedicated rib series compared with CT. Our results showed that even with optimized technique radiography seems to be inferior in detection of rib fractures compared to CT. Sensitivity of ribs series for the detection of rib fractures was 82%, while the sensitivity for revealing the correct number of fractures was only 73%. The differences were only statistically significant for detecting the correct number of fractures, probably because of the small study group. Rib series visualized more fractures than the plain chest films with a sensitivity of only 46%. Still the differences were not statistically significant. On the other hand the sensitivity of plain chest film compared to thorax CT was significantly inferior in the detection of rib fracture. Rib series but also plain chest film showing a very high specificity of 100%, and high inter-rater reliability. Based on the chart review we could exclude additional trauma in the delay period between CT and RS.

Hong et al. compared 56 postmortem pediatric thorax CT and radiographic examinations with the results of the coroner reports in cases of suspected child abuse. Sensitivity of CT was reported to be 85% compared to 46% of chest radiography [14]. Still the CT sensitivity for children and adults are not comparable, because of the different diameters of adult and non-adult ribs resulting in different detectability by CT.

Our results for the chest film are comparable to other published studies. The age and gender distribution is also comparable to the available literature data, which shows a majority of male patients.

Isolated fractures of the ribs without associated complication even with an observable dislocated fracture are being treated symptomatically [16]. Because of this fact the question arises, where the general medical use of a radiological documentation of rib series is. This question is being discussed in the literature controversially. It seems that the plain chest film provides more clinically relevant information because it can depict associated complications. If these complications are being excluded it doesn´t seem mandatory to prove that the patient has a rib fracture, especially if a rib contusion will led to the same therapeutic algorithm. An exception might be evaluations for forensic expert reports, because a radiographically proven rib fracture is a strong indicator for a thorax trauma. In cases of suspected child abuse the detection of bony lesions also plays an important role [17].

Of the 16 correctly identified patients with one or more rib fractures the numbers of fractured ribs were not found correctly in to two cases. This may lead to another problem of radiographic evaluation of minor thorax trauma because literature shows correlation between the number of involved ribs and complications with typical morbidity. The critical margin seems to be more than two ribs. For these patients rates of complications like hemato- or pneumothorax of 75% have been reported [16,18].

In our study only two of 22 patients with rib fractures had also a pneumothorax. Because of the low number a correlation is not possible. Another disadvantage of radiographic evaluation is that instead in CT no costal cartilage injury can be visualized [12].

At this point it is important to mention ultrasound, which is a very helpful diagnostic tool for the detection of rib fractures, too. Due to its high spatial resolution it may be even superior to CT. Griffith et al. revealed in their study with 50 patients a sensitivity of 90% for the detection of rib fractures, while radiography had only a sensitivity of 15%. Specificity of both modalities was 100% [13]. On the other hand, ultrasound suffers from known limitations such as high examiner and patient dependence, and no unique standards to perform and document examinations. MRI may also constitute a helpful tool in evaluating traumatic lesions of the bony part of the chest wall, but no data have been published so far. The limited availability is probably the limiting factor for its use in the diagnostic work up of emergency patients.

### Limitations

The retrospective design and the comparatively small number of included cases are the major limitations of this study. However, it is difficult to find patients who received both, RS and CT for the diagnostic work-up of minor thorax trauma.

## Conclusion

Our results suggest that RS for detecting rib fractures has lower sensitivity comparing to CT but our results were only significant for detecting the correct number of fractures, probably because of the small number of patients. Comparing with the results of PCF, dedicated radiography of the ribs provides higher diagnostic accuracy, but the differences were not significant. Due to the limited additional diagnostic information, RS seems not to be a useful examination in evaluation of minor thorax trauma, neither alone nor in combination with PCF. Method of choice to detect a rib fracture seems to be CT, but we have to be aware about the higher costs and radiation dose comparing to rib series. For these reasons we need further investigation of the clinical impact of the radiological proof of a rib fracture in minor thorax trauma by follow up studies with larger patient numbers.

## Article Summary

### Why is this topic important?

Minor thorax trauma is a common clinical condition which can cause rib fractures, pneumothorax and hematothorax. Although conventional radiography is considered to be of low diagnostic accuracy, rib series is a frequent examination in patients with minor thorax trauma.

### What does this study attempt to show?

This is the first systematic study comparing the diagnostic value of dedicated rib series, plain chest film and computed tomography of the chest for evaluation of minor thorax trauma.

### What are the key findings?

Rib series does not significantly outmatch plain chest film examination in the diagnosis of rib fractures.

In comparison to plain chest film rib series cannot provide any additional information such as the presence of pneumothorax of hematothorax.

Computed tomography is superior to rib series concerning the identification of the correct number of fractured ribs.

### How is patient care impacted?

Based on our results rib series cannot be recommended in the diagnostic work-up of patients with minor thorax trauma.

Instead of rib series, plain chest film should be performed for diagnosis of rib fractures and to rule out pneumothorax and hematothorax.

If the accurate number of fractured ribs is of clinical importance, an additional computed tomography of the chest should be performed.

## Consent

Written informed consent was obtained from the patient for the publication of this report and any accompanying images.

## Competing interests

The authors declare that they have no competing interests.

## Authors’ contribution

PH and AS designed the study. PH, CD, MW and AS carried out data analysis and interpretation. PH,CD and AS read the radiographics. MW and SS performed the statistical analysis. PH, LD and CS drafted the manuscript. All authors read an approved the final manuscript.
